# IPCO: Inference of Pathways from Co-variance analysis

**DOI:** 10.1186/s12859-020-3404-2

**Published:** 2020-02-18

**Authors:** Mrinmoy Das, Tarini Shankar Ghosh, Ian B. Jeffery

**Affiliations:** 1 APC Microbiome Ireland, University College Cork, Cork, Ireland; 2 School of Microbiology, University College Cork, Cork, Ireland

**Keywords:** Microbiome, Prediction, Functionality, Co-variance, Novel method

## Abstract

**Background:**

Key aspects of microbiome research are the accurate identification of taxa and the profiling of their functionality. Amplicon profiling based on the 16S ribosomal DNA sequence is a ubiquitous technique to identify and profile the abundance of the various taxa. However, it does not provide information on their encoded functionality. Predictive tools that can accurately extrapolate the functional information of a microbiome based on taxonomic profile composition are essential. At present, the applicability of these tools is limited due to requirement of reference genomes from known species. We present IPCO (Inference of Pathways from Co-variance analysis), a new method of inferring functionality for 16S-based microbiome profiles independent of reference genomes. IPCO utilises the biological co-variance observed between paired taxonomic and functional profiles and co-varies it with the queried dataset.

**Results:**

IPCO outperforms other established methods both in terms of sample and feature profile prediction. Validation results confirmed that IPCO can replicate observed biological associations between shotgun and metabolite profiles. Comparative analysis of predicted functionality profiles with other popular 16S-based functional prediction tools showed significantly lower performances with predicted functionality showing little to no correlation with paired shotgun features across samples.

**Conclusions:**

IPCO can infer functionality from 16S datasets and significantly outperforms existing tools. IPCO is implemented in R and available from https://github.com/IPCO-Rlibrary/IPCO.

## Background

Microbiome research has expanded exponentially over the last decade and has shown that microbiota communities have significant roles in health maintenance, as well as being key inputs into food and industrial processes [1, 2]. The study of microbiome communities fundamentally falls under two strategies: the taxonomic composition is determined by either amplicon sequencing (16S marker gene) or metagenomic whole genome shotgun sequencing (mWGS) with the latter providing additional information on the functional capabilities which allows the identification of genes and pathways. Despite the availability of mWGS, amplicon sequencing still remains popular due to its relatively low cost, quicker computation time, lower disk space requirements, and ability to detect a diverse set of taxa, including those with a low abundance based only on the marker gene. Reviews of these two approaches have discussed both the advantages and disadvantages of these methods [3, 4].

Amplicon sequencing is limited to providing only taxonomic information of the microbial communities. A number of tools can be used to predict the functional potential of the microbial communities obtained from 16S sequencing, the most cited being PICRUSt which was published as early as 2013 [5]. Other widely used tools that were developed later are Tax4Fun (2015) [6], and Piphillin (2016) [7]. All of these tools rely on functionally annotated reference genomes. The difference between them is the methodology used to map the amplicon data to these references and the approach used to assign functional annotation when suitable reference genomes are not available. PICRUSt works by considering the phylogenetic tree and the distance to the closest functionally annotated reference microbe [5]. It relies on the GreenGenes database [8] for matching the references to the queried amplicon data. The major limitation of PICRUSt comes forth in the case of 16S sequences, which do not have sequenced/annotated genomes of phylogenetically close relatives in the reference database. Tax4Fun and Piphillin implement BLAST and global alignment respectively between the amplicon data and the reference genomes obtained from different databases [6, 7]. All of these tools use KEGG orthologs (KOs) [9] annotation from reference genomes which is combined with amplicon abundance to predict the functionality. The limitation of all these methods is the requirement of sequenced/annotated reference genomes.

To overcome constrains of limited reference taxa, an alternative approach would be to use pairs of 16S amplicon and metagenomic datasets which show co-variance between the samples based on taxonomic abundance and functionality respectively. The identified co-variance trends can then be combined with the taxonomic abundance of the queried dataset for whom the functionality will be predicted. In this paper, we present IPCO, a tool based on this novel approach for inferring functionality of a 16S amplicon dataset. The primary advantage of our method is that it does not depend on the presence of sequenced and annotated genomes directly. IPCO is an application where values are assigned as the functional profiles for the samples of a 16S amplicon dataset based in a double co-inertia analysis involving the RLQ method (R-mode; Q-mode; and L-link between R and Q) [10, 11] between a paired taxonomic and functional dataset and a queried 16S amplicon dataset for which functionality will be inferred. Co-inertia analysis measures the concordance between two datasets, and maximises the squared covariance projected by two datasets [12, 13]. In paired taxonomic and functional profile datasets one would expect that alterations in the taxonomic profiles naturally should also reflect changes in its functional potential. Co-inertia can be further extended by application of the RLQ method, which integrates a third dataset (amplicon dataset in this case) and therefore analyses the co-inertia of the three datasets simultaneously. This methodology can provide a set of scores for the functional dataset and the amplicon dataset weighted by the paired taxonomic dataset.

IPCO’s performance is compared with PICRUSt, Tax4Fun and Piphillin in terms of both sample and feature correlation with KEGG pathways from experimental datasets. IPCO also predicts MetaCyc pathway profiles and these predictions are validated against a paired mWGS dataset. Correlation of mWGS functional profiles against paired bile acids and short chain fatty acids (SCFAs) metabolite profiles confirmed the metabolomic associations with the observed metagenomic pathways. IPCOs ability to reproduce these biological associations is validated against these observed biological associations.

## Results

### Description of the study cohort

In the current study, Table 1 describes the cohort retained after removing samples with low sequencing depths and stratified functional features for the initial analysis. The samples retained were investigated using the IPCO, PICRUSt [5], Piphillin [7] and Tax4Fun [6] to evaluate the performance of the tools.

**Table 1:** Number of mWGS features and samples retained in the initial analysis

	Samples	KOs	KEGG pathways	MetaCyc	OTUs
HMP nasal	61	5971	129	659	7464
HMP oral	71	5829	133	625	13,696
HMP skin	8	4886	132	593	3492
HMP stool	87	6356	128	712	9966
Water (Brazil’s river)	37	2724	114	497	1185

Total number of samples and features across the five reference datasets

IPCO was initially implemented in the HMP [14] stool cohort to investigate the effects of different data transformation, taxonomic levels and sample size thresholds.

### Overview of IPCO implementation

IPCO is dependent on paired reference datasets comprising of taxonomic and functional abundances. To ensure that the inferred functionality of a query dataset is not due to overfitting or homogeneity among reference and query profiles, a bootstrap methodology was implemented to ensure that the reference and query datasets do not contain the same samples. The dataset at each site was randomly split as described in the methods. This split ensures that samples in the reference are not present in the query. This was carried out with 100 iterations to ensure that the result is unbiased for each analysis. The correlation values (sample-to-sample and feature-to-feature) between inferred and mWGS functional profiles was calculated at each iteration and the average was taken across the 100 iterations.

### Data transformation of the reference datasets affects IPCO results

In IPCO, the covariance between the three datasets (R, L and Q) was observed to vary depending on the transformation and normalisation method used. Investigation with the transformation/normalisation mentioned in the methods is shown in supplementary Fig. 1, Additional file 1 and supplementary Table 1, Additional file 2. The Hellinger transformation was observed to have a higher RV coefficient for both samples and features and similar correlation values compared to other methods. Observed RV coefficients were significant (*p*-value ≤0.05) for all cases. The Hellinger transformation is implemented as default for all further analysis.

### Lowest taxonomic levels and high samples size provides best results with IPCO

**Fig. 1:**
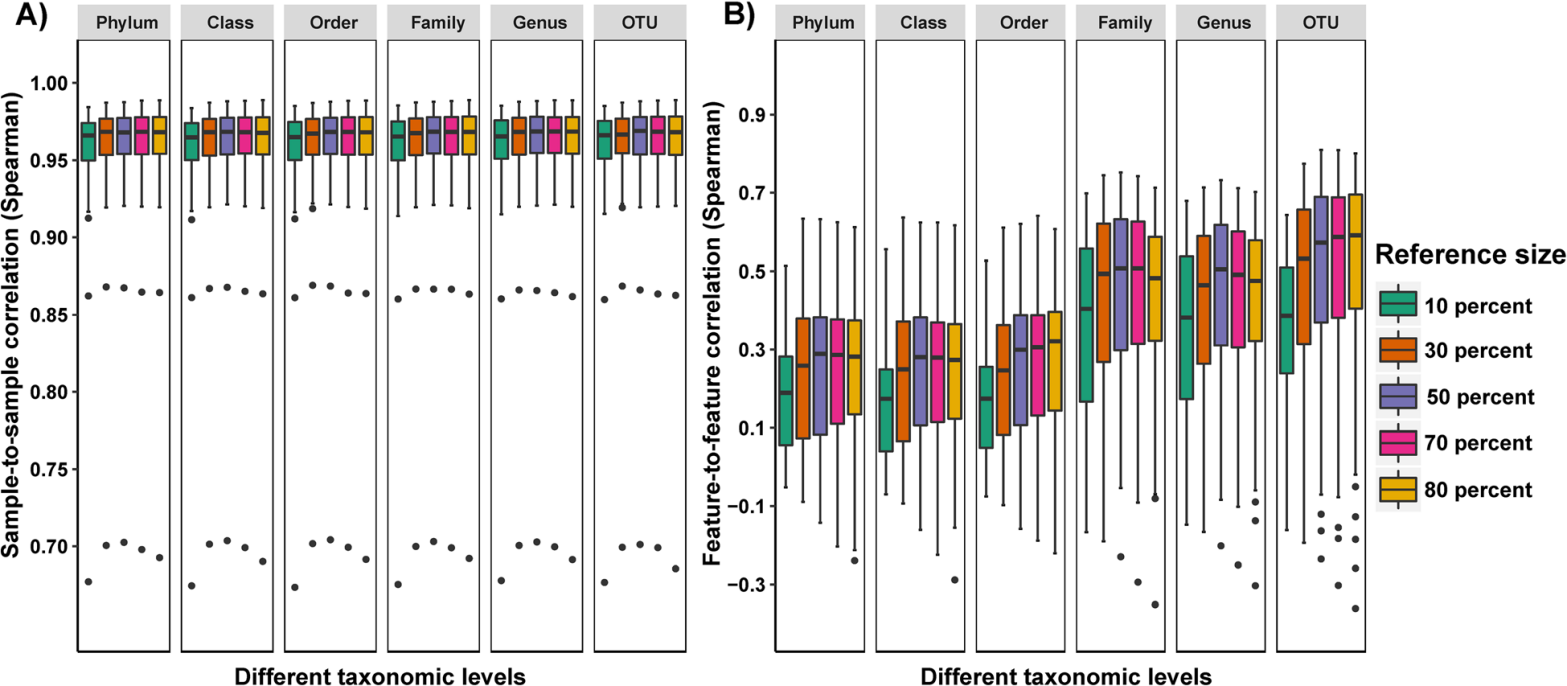
Sample and feature correlation using KEGG pathway abundance at different taxonomic levels and reference dataset size. Boxplots showing the variation of **a**) Sample to sample correlations **b**) Feature to feature correlations obtained between the inferred KEGG pathway abundances and the mWGS functional profiles at all different reference sizes and taxonomic levels

Implementation of IPCO with 100 bootstraps for each subsampling at different reference sample sizes on both KEGG and MetaCyc pathway abundance datasets showed that the best sample and feature correlations were observed with the lowest taxonomic levels and highest sample size (Fig. 1a-b, supplementary Fig. 2A-B, Additional file 1). No significant difference was observed in the sample correlation for any reference dataset size except for between 10% and other reference sizes at family level in the MetaCyc dataset **(**supplementary Table 2, Additional file 2**)**. However, the feature correlation increased with increased sample size and at the lowest taxonomy levels. No significant differences were observed for feature correlation using a reference size of at least 30% or larger in the KEGG pathway analysis and 50% or more for MetaCyc at the different taxonomic levels investigated **(**supplementary Table 2, Additional file 2).

The outlier observed in Fig. 1 and supplementary figure is due to increased abundances of pathways in that sample which are observed to be decreased in other samples. Looking at the KEGG pathways for the outlier samples, it was observed that the most abundant pathways also had a low coverage (maximum coverage = 1e-04).

The randomisation of mWGS samples through shuffling during evaluation at each iteration resulted in a complete lack of feature-to-feature correlation (supplementary Fig. 3**,** Additional file 1). Although a high sample-to-sample correlation was observed due to the functional redundancy across samples, the pattern associated with different thresholds and with the unshuffled predictions seen in Fig. 1 and supplementary Fig. 2 were lost.

### IPCO outperforms PICRUSt, Tax4Fun and Piphillin in terms of both sample and feature correlation

**Fig. 2:**
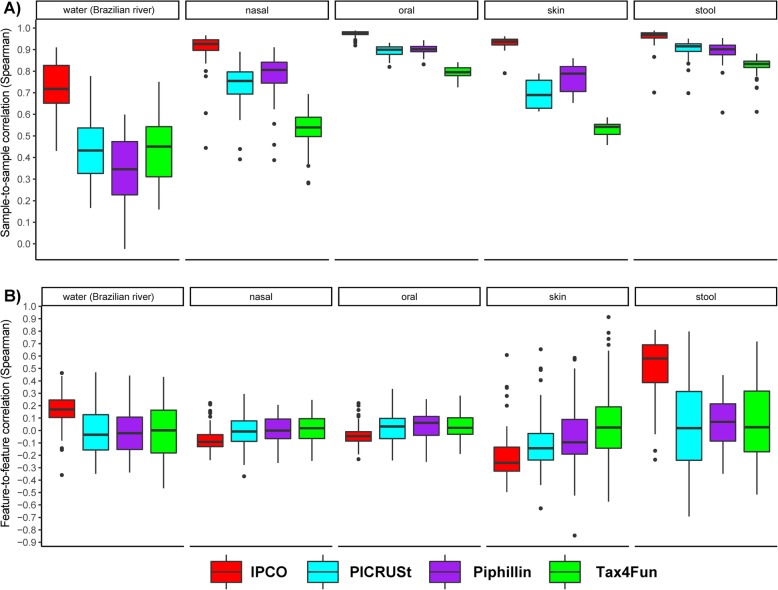
Sample and feature correlations between inferred KEGG pathways and mWGS KEGG pathways profiles at different sites and using different methods. Boxplots showing the comparison of **a**) Sample to sample correlations and **b**) Feature to feature correlations obtained between the inferred KEGG pathway abundance and the mWGS functional profiles at different sites using different methods

To evaluate the functional inference of IPCO, we applied PICRUSt, Tax4Fun and Piphillin to the same datasets (Table 1) and KEGG pathway profiles were inferred. Spearman correlation was calculated for the inferred pathway profiles against its mWGS abundance both in terms of sample and feature correlation. IPCO outperformed PICRUSt, Tax4Fun and Piphillin in terms of sample correlation across all datasets (Fig. 2a**,** supplementary Table 3, Additional file 2). IPCO showed highest sample correlation with a narrow IQR range for stool and oral samples. Skin and nasal dataset showed lower sample correlation compared to stool and oral, however it was observed to be higher than what was observed using the other tools. The lowest sample correlation was observed using the Brazilian river water dataset, but it was also higher than other tools for that site (supplementary Table 3, Additional file 2).

Upon investigating the feature-to-feature correlations, it was observed that IPCO outperforms PICRUSt and Tax4Fun in stool and Brazilian river water datasets (Fig. 2b). Nasal, oral and skin dataset revealed a lack of correlation using IPCO. It was noted that across all datasets, the median feature correlation for PICRUSt, Tax4Fun and Piphillin was close to zero.

**Fig. 3:**
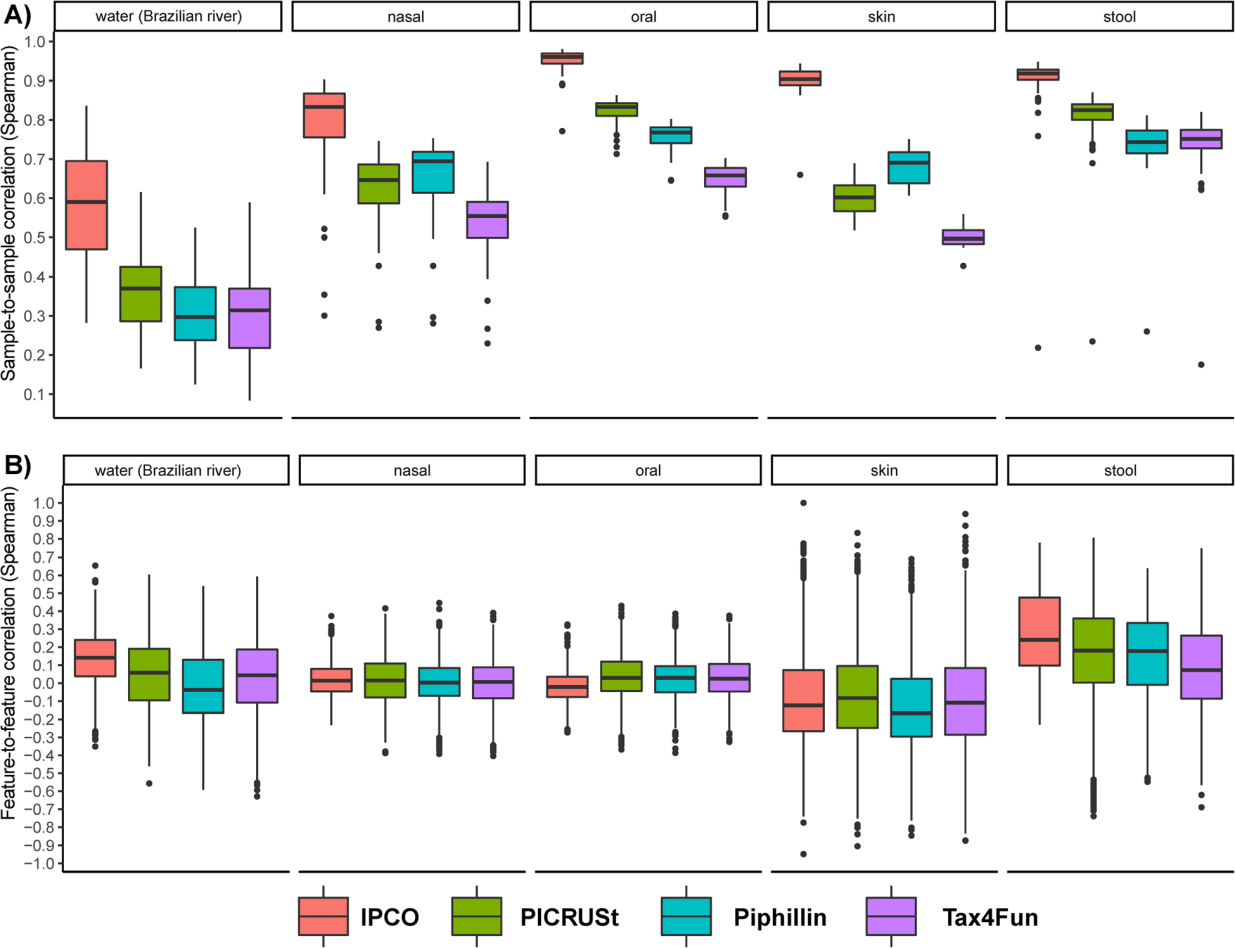
Sample and feature correlations between inferred KO and mWGS KO profiles at different sites and using different methods. Boxplots showing the comparison of **a**) Sample to sample correlations and **b**) Feature to feature correlations obtained between the inferred KOs and the mWGS KO profiles at different sites using different methods

Sample-to-sample and feature-to-feature correlations based on KO abundances obtained from IPCO, PICRUSt, Tax4Fun and Piphillin were also calculated. IPCO was observed to outperformed other methods in terms of correlation values between both inferred sample and feature profiles when compared against the observed mWGS sample and feature profiles (Fig. 3**,** supplementary Table 4, Additional file 2) for faecal and Brazilian river water datasets. Feature-to-feature correlations for the remaining sites (nasal, oral and skin) were poor with the median correlation being close to zero for all tools including IPCO.

The IPCO inferred KO profiles obtained from the HMP stool dataset using the equal reference to test split (50:50) was further processed to obtain the inferred-KO KEGG pathway profiles at each iteration. Interestingly, these profiles at pathways level showed a higher feature-to-feature correlation (1st quartile: 0.31, median: 0.49, 3rd quartile: 0.67, supplementary Fig. 4) when compared to other published tools but lower than the default IPCO methodology as observed in Fig. 2.

### Lack of significant covariance between taxonomy and functional datasets results in a lack of predicted feature correlation in nasal, oral, skin, and Brazilian river water datasets

To investigate the poor performance by IPCO on the other sites excluding stool, the co-variance between the taxonomic and functional dataset was calculated. Co-inertia analysis of the taxonomic profiles with its paired functional datasets across all sites revealed a lack of significant covariance between taxonomy and functional profiles in the nasal, oral and skin datasets (supplementary Table 5, Additional file 1). The Brazilian river water dataset showed significant covariance when the mWGS derived taxonomy was used (while the 16S dataset did not co-vary significantly) (supplementary Table 5, Additional file 1). This may in part explain the poor performance of IPCO on these datasets. The taxonomic abundance of the reference dataset is not reflected in its paired functional dataset which resulted in a lack of significant covariance. It was observed that the functional diversity determined by the observed number of pathways had a narrow range compared the paired taxonomic diversity which showed more variation for all body sites excluding the stool dataset. This functional redundancy and the lack of detection of unique functionality suggests that the functional heterogeneity of species was not accurately reflected in these datasets. Given the lack of covariance in these datasets, further analysis was carried out using the stool dataset.

### Higher pathway coverage improves functional inference

**Fig. 4:**
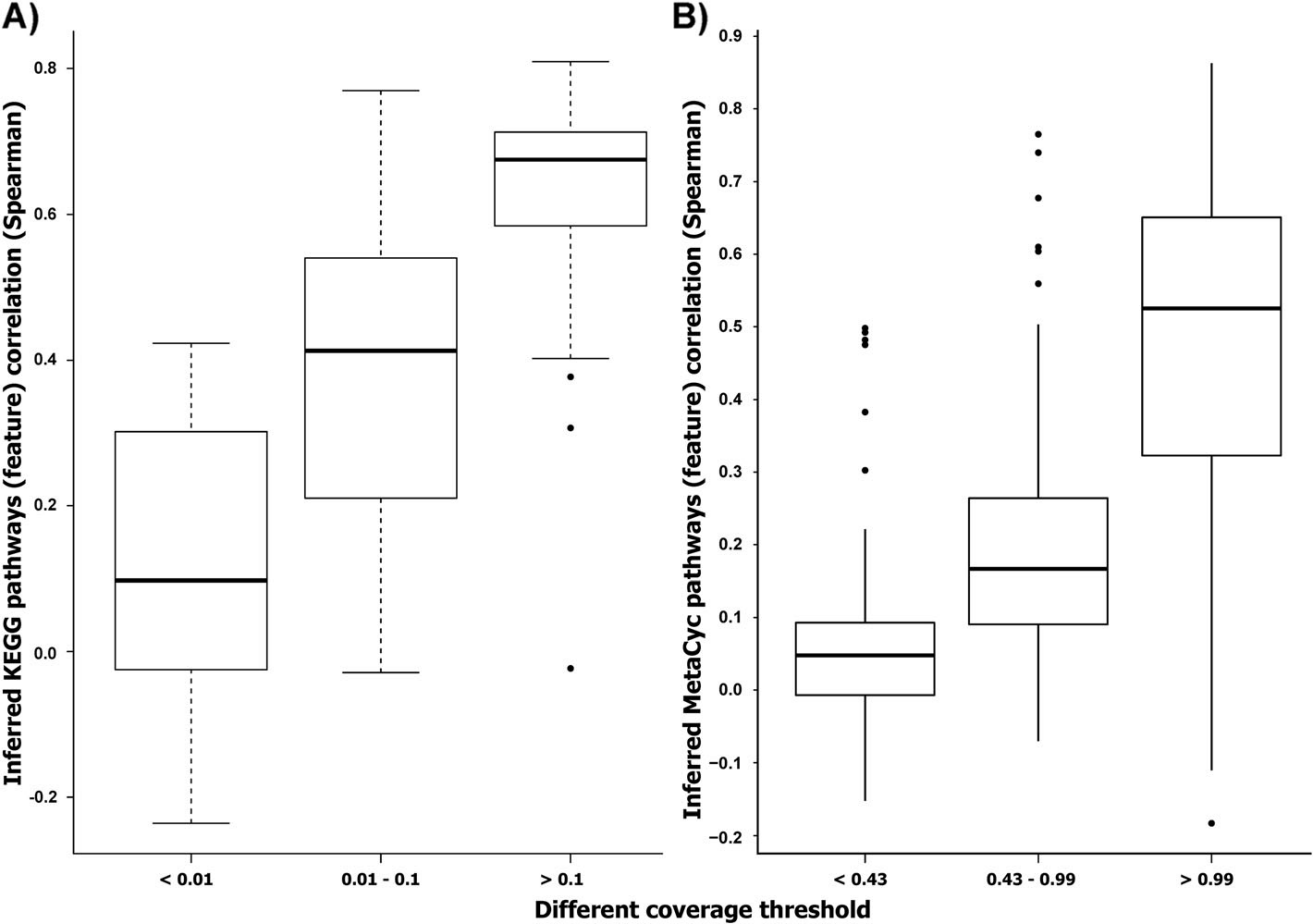
Comparison of feature-to-feature correlations at different pathway coverage thresholds for the KEGG and MetaCyc. Boxplot showing the functional feature-to-feature correlations obtained using different coverage thresholds for the **a**) KEGG pathways and **b**) MetaCyc pathways

Investigation of the effect of pathway coverage on the correlation values between the observed pathway abundance and the inferred pathways obtained using IPCO showed that coverage correlated well with functional pathway prediction (Fig. 4a). Based on this, the KEGG pathways were binned based on thresholds such that pathways below the mean coverage of 0.01 were considered low correlation predictions and pathways with a mean coverage over 0.1% were considered high correlation predictions with the remaining pathways with a mean coverage between 0.01 and 0.1 being considered medium correlated predictions. These predictions showed a correlation value between 0.25–0.6 whereas the high correlated predictions had correlation values between 0.6–0.7 (Fig. 4a). In case of MetaCyc pathways, we observed similar results where pathways with coverage less than 0.41 (1st quartile of mean coverage across samples) were low correlated predictions. Pathways whose mean coverage was between the 1st quartile (0.41) and the median value (0.99) showed improved feature correlation for the inferred pathways whereas the best feature correlation (0.37–0.62) was observed for those pathways whose average pathway coverage was greater than its median value (Fig. 4b). For both KEGG and MetaCyc, we were able to get high correlation values for more than 50% of the pathways based on the coverage filtering. The number of pathways binned into each of the coverage thresholds for both KEGG and MetaCyc are described in Table 2. All reported observations were carried out on HMP stool functional datasets.

**Table 2:** Pathways retained at different coverage thresholds

KEGG coverage threshold	Total	< 0.01	0.01–0.1	> 0.1
Number of KEGG pathways	118	19	30	69
MetaCyc coverage threshold	**Total**	**< 0.43**	**0.43–0.99**	**> 0.99**
Number of MetaCyc pathways	693	173	166	354

Number of KEGG and MetaCyc pathways identified at different coverage thresholds. Coverage thresholds are strongly correlated to the accuracy of the inferred pathway profiles

The mean coverage for the predicted KEGG pathways from PICRUSt, Tax4Fun and Piphillin modelled against its feature correlation values showed no positive association between pathway coverage and feature correlation (supplementary Fig. 5, Additional file 1). This indicates that the coverage filter was not applicable for these tools as opposed to IPCO. All subsequent analyses were carried out using all the functionalities without filtering for coverage.

### IPCO can accurately infer sample and feature profiles using taxonomy from mWGS or 16S amplicon datasets

An alternative approach to using a reference 16S dataset and paired mWGS functional is to derive the taxonomic information from the reference mWGS dataset itself. Investigation of using taxonomic information derived from mWGS showed a comparable performance to using a 16S species or closed OTU level dataset when inferring the functionality of an external 16S dataset (supplementary Fig. 6, Additional file 1). Table 3 describes the sample characteristics considered for this analysis. The reference dataset in this case consists of a large cohort of healthy samples as detailed in the methods (Ghosh et al. Submitted). The validation dataset used is the ELDERMET dataset [15].

**Table 3:** Number of samples, pathways, and species in the validation datasets

	Samples	KEGG pathways	MetaCyc	mWGS Species	16S Species	Closed OTU
Validation	79	123	776	NA	201	842
Reference healthy	1180	143	833	772	NA	NA
HMP	87	128	712	353	282	1341

The number of samples and features present in the reference healthy and validation (ELDERMET) datasets, which were used when inferring functional profiles and comparing with observed functional profiles of the validation dataset. NA; Not applicable

It was observed that the use of species levels datasets obtained from the same mWGS data that was used to compute metagenomic functional profiles was sufficient to infer functionality for 16S datasets (supplementary Fig. 6, Additional file 1). Further validation involving the replication of biological pathway to metabolite associations was carried out using the reference healthy functional and paired mWGS species profiles as reference in IPCO to infer the functionality of the ELDERMET 16S dataset.

### Healthy references implemented with IPCO shows better inferences for diseased samples

**Fig. 5:**
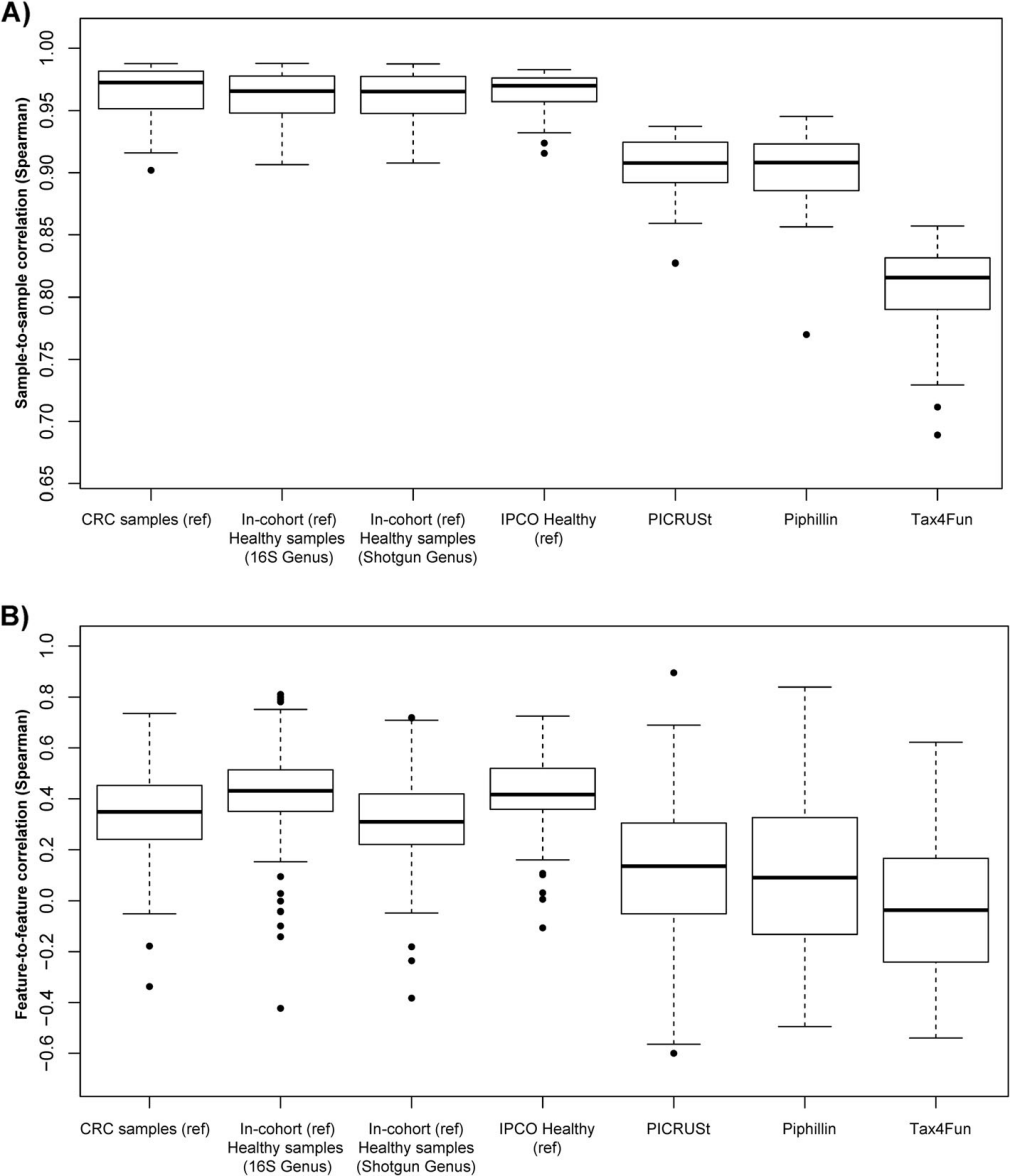
Comparison of sample and feature correlation observed when using different reference datasets to predict CRC sample functionality (KEGG pathways). Boxplot showing **a**) Sample-to-sample correlation observed with the use of only CRC samples as reference, healthy samples from same cohort as reference where the reference taxonomic dataset is 16S genus profiles, healthy samples from the same cohort with mWGS genus profiles as reference taxonomy, IPCO reference healthy at genus level (taxonomy reference), PICRUSt, Piphillin and Tax4Fun. **b** represents feature-to-feature correlation observed with the use of different references as described for Fig. 5a

IPCO was implemented with both in-cohort and external healthy references on the CRC samples (16S genus level profiles) from the Zeller et al. The predicted pathway feature from the CRC dataset highlighted that the healthy references can be used to predict the diseased samples as determined by the high sample and feature correlation observed (Fig. 5, supplementary Fig. 7, Additional file 1, supplementary Table 6, Additional file 2). The healthy IPCO reference and in-cohort healthy reference samples resulted in higher predicted feature correlation (KEGG predicted CRC vs mWGS CRC) compared to using CRC dataset as reference itself. Repeating the analysis using MetaCyc, the healthy reference (in-cohort 16S genus profiles) showed higher feature correlation compared to using CRC as reference but there were no significant differences in the sample profile predictions.

### Inferred profiles from IPCO replicate mWGS functional pathway to metabolite profile associations

Correlation of the mWGS derived functionality (KEGG and MetaCyc) from ELDERMET samples to paired bile acid and SCFA profiles identified associations between key pathways and biologically relevant metabolites.

Investigation of the bile acid profiles showed that KEGG pathway “ko00121: Secondary bile acid biosynthesis” pathways significantly negatively correlated with primary bile acids (cholic acid and chenodeoxycholic acid) while “ko00790: Folate biosynthesis” known to promote bile acid levels [16] is observed to be significantly positively correlated with primary bile acids in ELDERMET mWGS data.

With the secondary bile acids (lithocholic acid, dehydrocholic acid, 12-ketolithocholic acid, dehydrolithocholic acid, hyodeoxycholic acid and isolithocholic acid), it was observed that “ko00121: Secondary bile acid biosynthesis”, “ko00430: Taurine and hypotaurine metabolism”, “ko03070: Bacterial secretion system”, “ko05100: Bacterial invasion of epithelial cells” were all significantly positively correlated with secondary bile acid levels. This validates the bile acid profiles and the ELDERMET mWGS functional dataset profiles in accordance to actual biochemical mechanism observed in a bacterial system.

**Fig. 6:**
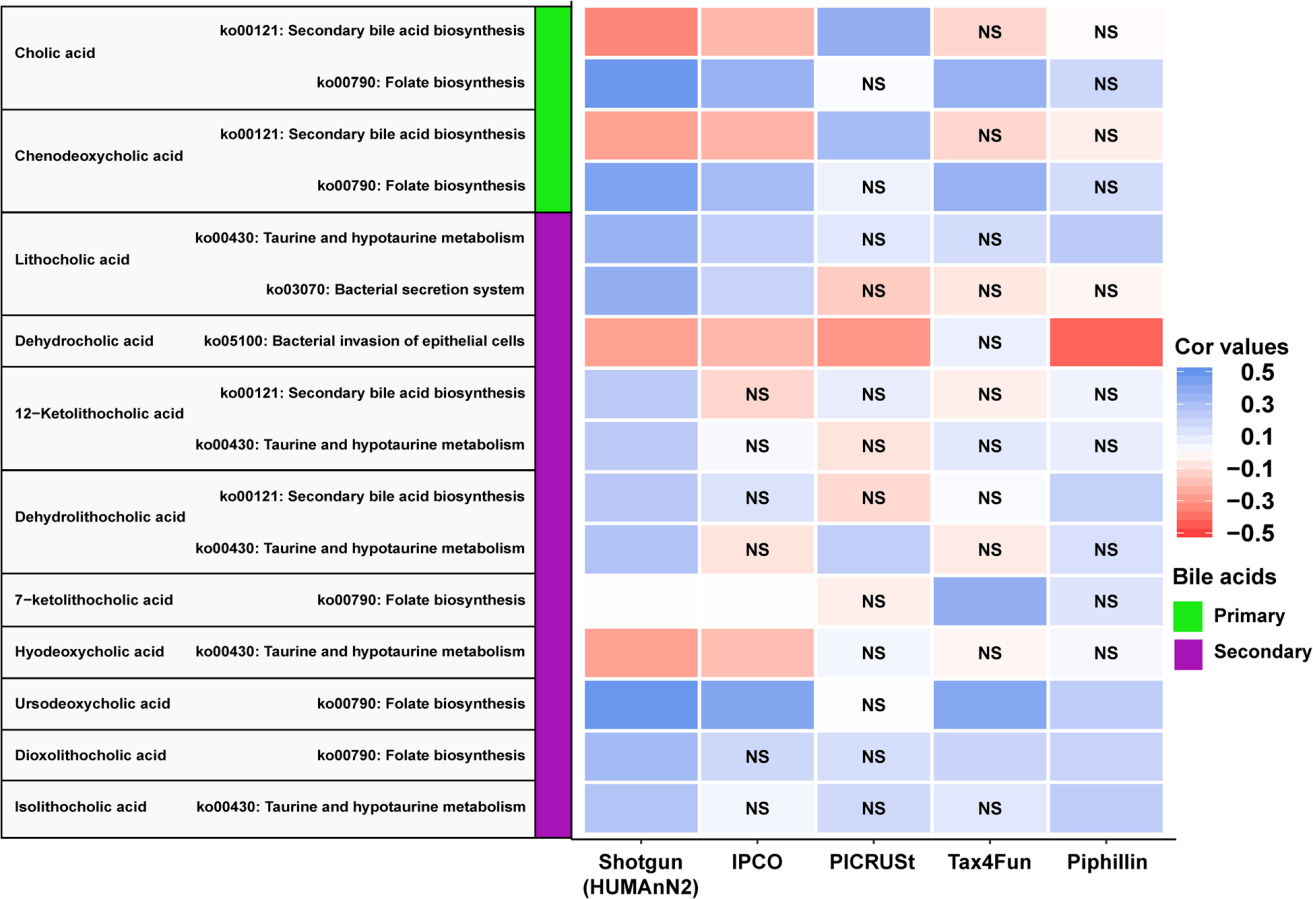
Correlation of the bile acids metabolite profiles with inferred KEGG pathway abundances from various methods. Correlation of the inferred bile acid metabolite profile to paired mWGS KEGG pathways shows significant association (p-adjusted ≤0.1) with known pathways as shown in 1st column. Directionality of association is shown by correlation values colour intensity. Pathways inferred from IPCO shows same directionality and significance (*p*-value ≤0.1) as observed with mWGS profiles for most cases. “**NS**” inside the cell represent non-significant (*p*-value > 0.1) associations

The results of the inferred functional profiles obtained from all the tools showed that IPCO provides the best estimation of the observed associations between the mWGS dataset and bile acid profiles (Fig. 6**,** supplementary Table 7, Additional file 2). It was observed that for primary bile acids only PICRUSt showed significant correlation with “ko00121: Secondary bile acid biosynthesis”, however the directionality was reversed. Neither Tax4Fun nor Piphillin showed significant associations for “ko00121: Secondary bile acid biosynthesis” abundance and primary bile acids. Looking at the secondary bile acids, we observed that 12-ketolithocholic acid did not show any significance with the inferred profiles obtained from all tools. Lithocholic acid and KEGG pathways obtained from IPCO agreed with mWGS results. Tax4Fun was significant but showed the opposite directionality to the observed association. Dehydrolithocholic acid was significantly associated with KEGG pathways in PICRUSt and Piphillin. All associations, directionality and significance from all tools compared to mWGS results are highlighted in Fig. 6. Overall, while correlating measured bile acids levels to predicted KEGG pathway profiles, IPCO was successful 62% of time considering the directionality and statistical significance. PICRUSt, Tax4Fun and Piphillin were correct only 12, 31 and 38% of time respectively.

Similar results were observed when the MetaCyc pathway abundance dataset was used as reference. Correlation of the ELDERMET mWGS MetaCyc pathways with bile acid profiles show significant correlation with “PWY-6518: glycocholate metabolism (bacteria)” and “1CMET2-PWY: N10-formyl-tetrahydrofolate biosynthesis”. These results were replicated with the inferred MetaCyc pathway profiles obtained from IPCO (supplementary Table 8, Additional file 2).

The correlation between ELDERMET KEGG pathway abundance and SCFA (butyrate and propionate) profiles were observed to be not significant which included butanoate and propanoate metabolism, protein and amino acid metabolism (Lysine, Glumatine) pathways. This lack of association is consistent with the literature [17] and was replicated in the inferred profiles obtained from IPCO and PICRUSt (supplementary Table 9, Additional file 2). However, Tax4Fun and Piphillin showed significant associations for the inferred KEGG pathways obtained using those two tools for both butyrate and propionate levels. These significant associations are considered false positives, as they were not observed with the mWGS data. Piphillin reported the highest number of false positive pathways for butanoate. In the case of propionate, IPCO also predicted two false positive pathways.

Investigation of the SCFAs (butyrate and propionate) levels with ELDERMET mWGS MetaCyc pathways replicated the KEGG analysis and again showed that butyrate shows no significant association with key butyrate MetaCyc pathways after adjustment as is consistent with the literature [17]. This observation was replicated in the IPCO inferred functional profiles (supplementary Table 10, Additional file 2). Propionate also showed no significant association with mWGS pathways and this observation was replicated with IPCO inferred pathways (supplementary Table 10, Additional file 2). As other tools do not report MetaCyc pathway profiles, this investigation could not be carried out with other tools.

## Discussion

We have developed IPCO, a novel tool which predicts functionality for 16S amplicon datasets but is not dependent on the direct mapping of 16S sequences to known reference genomes. Instead, it utilises paired mWGS functional and taxonomic datasets as references that are built from annotated genomes but do not assume that the functional potential of the taxa is the same as the reference genome.

Alterations at taxonomic level will affect the overall functional potential at community level [18]. The robustness of the association between functionality and taxonomy is dependent on both the abundance of the various taxa and the distribution of function across these taxa. Using this concept, IPCO, is able to utilise the biologically and statistically significant covariance observed between the reference taxonomic and functional datasets and infer the functional capabilities of an external 16S amplicon dataset. The IPCO implementation is also unique in that it provides a distinction between high quality predictions and lower quality predictions based on pathway coverage for both KEGG and MetaCyc pathways.

IPCO is reliant on the availability of reference datasets from the environment being studied. This raises the question of the appropriateness of using a set of defined samples (e.g. healthy reference) to infer samples that are dissimilar in some aspect (e.g. diseased) even within the same environment. By using different types of reference samples, it is observed that the healthy samples may be used to predict diseased samples as long as they are obtained from the same environment. In fact, in this analysis, the functional inference capability of IPCO for both KEGG and MetaCyc was better with the use of healthy samples from the same site (Fig. 5**,** Supplementary Fig. 7).

One weakness of available functional prediction tools is that using a reference set of known functions from only a set of known taxa limits the prediction of functionality for the amplicon dataset. This limitation results in a lack of feature-to-feature correlation i.e. KEGG pathway abundance calculated from 16S datasets do not correlate well across samples that are obtained from similar environments when compared with a paired mWGS dataset for the same samples. This is of concern as this potentially creates false positive results and/or reversed directionality when investigating functional profiles inferred from 16S datasets. By studying the IPCO inferred functionality and associations with biologically relevant metabolites, we have shown that the inferred functional capabilities obtained using IPCO, mimics the results of the mWGS functional profiles and outperform other predictive tools.

The filtering criteria in IPCO allows the users to select a set of functional pathways with sufficient coverage to be inferred. This removes functional pathways which may have been spuriously assigned due to the presence of only a small subset of genes/reactions. It is noteworthy that despite using a uniform method to tabulate the pathway level information for all the tools, the pathway coverage information could not be used to improve predictions by filtering out low-coverage pathways for any tools except IPCO. The lack of association between feature coverage and correlation observed in other tools may be due to the assumptions made when mapping to functionally annotated reference genomes by the published tools. The reproducibility of results observed from both KEGG and MetaCyc shows that our method is independent of the functional annotation used and so may be implemented with custom datasets built from user’s internal data. Currently, IPCO can be implemented with any taxonomic level information and the taxonomic assignment can be done with any reference database as long as the taxa are present in the reference which acts as a mediator to co-vary the functional profiles with the 16S dataset. This may allow alternative implementations of the IPCO methodology such as extrapolation of functional information for a set of samples obtained from shallow sequencing by using a subset of samples with deeper sequencing depth.

Overall, IPCO had a superior performance compared to other tools using both KOs and KEGG pathways, even when inferred KO-KEGG pathways was used. The feature correlation observed across all tools including IPCO were lower using KEGG pathway level predictions. This could be potentially due to increased sparseness at KO level compared to pathway levels. The processing time also increases drastically due to the increased size of functional reference dataset (Number of KEGG pathways in HMP stool (*n* = 87) dataset is 128 whereas the number of KOs for the same dataset is 6356) which would be a limitation when using on a personal laptops or system with low computational and memory capacity. Similarly, the use of UniRef gene profile datasets is not feasible currently as nearly 2 million UniRef genefamilies are detected for the HMP stool samples alone which would require long processing times and would require a high performance computation system to process such a large dataset.

IPCO performs better than the other established tools but it is not without its limitations. IPCO is reliant on a paired mWGS functional and taxonomic reference datasets which rely on functionally annotated genomes. As with other tools, sample profile predictions will appear to be highly correlated to the actual sample profiles due to functional redundancy at the pathway level where highly abundant pathways are shared across multiple taxa. Therefore, care should be taken when interpreting the predicted sample profiles. IPCO assigns a small pseudo value to each functionality due to the way the R’LQ algorithm calculates double co-inertia, which makes the resulting inferred functionality a non-zero abundance. To overcome this limitation, the low abundant functionality can easily be filtered by removing those functions whose average inferred abundance across samples is below a certain quantile determined by the user. Although, other tools predicted the features poorly across all sites, IPCO also performed poorly on the non-gut samples. Our analysis showed that this was due to a lack of suitable reference datasets (lacking significant co-variance between reference taxa and functions), but IPCO can be easily tuned to work at other body sites or environments as suitable mWGS data from these different environments become available. This is noteworthy as inferring functionality using amplicon-based approaches rely on its concordance with the functional profile, which is not possible if the functional-level distribution is discordant with the taxonomy. Why this would be the case is beyond the scope of this manuscript. The use of IPCO is also limited to potentially only those environments, for which a reliable reference (i.e. significant covariance between taxonomic and functional profiles) is available. Samples from environments (e.g. low biomass) that may lack mWGS dataset limits the use of IPCO.

## Conclusion

IPCO provides a novel approach for functional inference, which is not directly dependent on the availability of functionally annotated reference genomes. The IPCO inferred functionality profiles reflect the true observed biological functionality. IPCO can be easily implemented with the default datasets or with in-house reference datasets without relying on the external reference datasets. Overall, IPCO provides a reliable inference of functional potential and can be easily implemented in the R statistical software.

## Methods

### IPCO algorithm

IPCO is an implementation of the RLQ analysis which is also known as fourth corner analysis. It requires a reference taxonomic and functional paired dataset along with a third dataset, which is the 16S dataset for which the functional potential will be inferred. RLQ analysis is a double co-inertia method which explores two datasets (R and Q) through a mediator dataset (L). IPCO implements RLQ to associate the functional profiles (R) with a 16S profile dataset (Q) which is the 16S dataset for which functions need to be inferred through a mediator taxonomic profile dataset (L). R and L datasets are related as they have the same samples (paired) and are used as reference datasets. Q and L datasets are related as they have the same taxa identifiers. Functional profiles of R and taxonomic profiles of Q are standardised and scaled through the weighted average where the weights of the samples and taxa are obtained from L dataset. Through RLQ methodology, we obtain a R’LQ product table, which an association matrix of R and Q mediated through L abundance. In IPCO, we re-standardise the R’LQ products by adding the weighted average of the functional potential back to the association matrix to obtain inferred functional profiles for the samples of Q dataset.

In summary, IPCO implements the following steps:

L = Matrix from correspondence analysis of reference taxa Table (L table).

R = Matrix from PCA of reference functional Table (R table) weighted by samples from L.

Q = Matrix from PCA of query taxa Table (Q table) weighted by taxa from L.

RLQ product = RLQ analysis (R, L, Q) (as described in the original paper).

rw = row weights from correspondence analysis of L.

waR = Weighted average of R table given by (∑R[, j] * rw) / ∑rw (note: removed from RLQ product in RLQ calculation).

where j represents 1 to n^th^ sample.

Inferred profiles = RLQ product [i,] + waR.

where i represents 1 to n^th^ feature.

### Data collection

In the current study, human microbiome taxonomic and functional profile datasets were obtained from the HMP project [14, 19] using the curatedMetagenomicData R library (v.1.10.0) [20]. mWGS functional (UniRef genefamilies) and taxonomic profiles datasets were obtained using the R library curatedMetagenomicData and paired V3-V5 16S rRNA OTU table was obtained from 16SHMPData R library (v.1.2.1) [21]. Paired datasets were obtained for nasal, oral (buccal cavity), skin and stool samples. Representative OTUs of V3-V5 regions were downloaded from the HMP website [14, 19].

A larger reference dataset consisting of functional and taxonomic profiles generated from only mWGS data were also obtained from the curatadMetagenomicData. This set is comprised of 1180 healthy samples from various cohort as described in Ghosh et al. (manuscript submitted).

Paired 16S and mWGS of an environmental dataset (Brazilian river water) used in this study are described in Tessler et al. [22] and downloaded from the NCBI SRA (PRJNA389803, PRJNA310230). This cohort comprised of paired 16S rRNA and mWGS data obtained from four major rivers in Brazil: Amazon, Araguaia, Paraná, and Pantanal. The 16S rRNA and mWGS sequences was quality filtered using Trimmomatic (v.0.38) [23]. Using USEARCH (v8.1), the quality filtered 16S rRNA sequences were dereplicated, clustered at 97% identity and chimera filtered (*de-novo* and using ChimeraSlayer) to obtain representative OTU sequences. Quality filtered reads were mapped to these OTUs to obtain the OTU table. The quality filtered mWGS data was processed using HUMAnN2 (v. 0.7.1) [24] to obtain mWGS derived taxonomic and functional profiles.

MetaCyc and KEGG pathway mapping files as provided with HUMAnN2 and HUMAnN1 were filtered to remove all known eukaryotic pathways. All samples from all datasets (UniRef gene profiles) were processed against the filtered MetaCyc mapping file to obtain the MetaCyc pathway abundance and coverage datasets.

The UniRef genefamilies dataset for all the samples were regrouped to KEGG Orthologs (KOs) IDs using humann2_regroup_table.py script and the KEGG to UniRef mapping provided in HUMAnN2 utilities. The regrouped KOs were processed using HUMAnN2 using the filtered HUMAnN1 KEGG pathways legacy database to obtain KEGG pathway profiles for the mWGS dataset.

All OTU datasets were filtered to remove samples with a sequencing depth of less than 1000 reads. Samples removed from OTU datasets were omitted from their paired mWGS datasets also. Normalised unstratified functional information was used in the implementation after the removal of UNMAPPED, UNGROUPED and UNINTEGRATED variables.

### Data normalisation and transformations

Taxonomic and functional abundance datasets were transformed using the following transformations: Z-scaling, proportion normalisation, log10 on rarefied and log10 on proportional data with 1e10^− 5^ added as minimum count value, Hellinger transformation [25] and centred log ratio (clr) transformation [26]. These transformations were investigated to identify the transformation best suited for IPCO.

### Validation of IPCO predictions

**Fig. 7:**
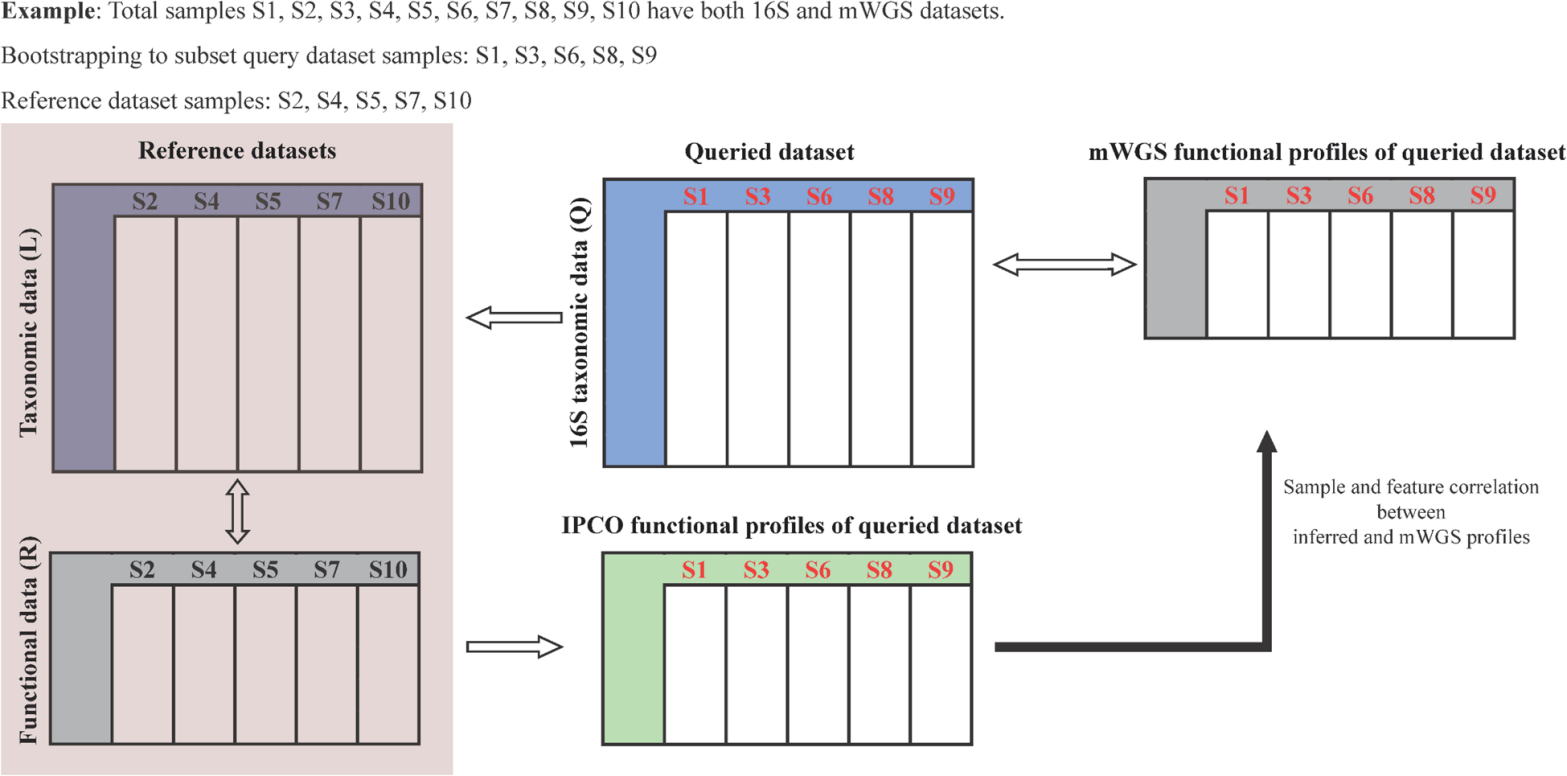
Implementation of IPCO and bootstrap iterations. The datasets were randomly subsampled into query and reference dataset. Reference dataset consist of taxonomic and functional profiles for the same samples. Reference taxonomic and queried dataset consist of different samples but are mapped to same taxa. The inferred profiles are correlated against the mWGS functional profiles for the query dataset to obtain the degree of associations. One hundred bootstrap iteration were carried out to randomly generate different subsamples and measure the degree of association for each iteration

IPCO is dependent on reference paired functional and taxonomic datasets. To validate the methodology, a bootstrap strategy was implemented to evaluate its predictions (Fig. 7). A subset of the samples from the 16S table were randomly selected and considered as Table Q. The samples omitted in Q formed the taxonomic Table L and were matched with its pathway abundance dataset to obtain a paired functional (R) and taxonomy (L) datasets thus removing pathway information for the samples present in Table Q. Using IPCO on R, L and Q table, pathway profiles were obtained for the samples from Q. Both inferred sample and feature (pathways) values were correlated using Spearman correlation with the actual mWGS pathway dataset for those samples present in Q. The bootstrapping analysis was repeated with 100 iterations to randomly subsample the reference datasets. An average was taken for both inferred sample to actual sample and inferred pathway to actual pathway correlation values from the 100 iterations.

### Effect of reference dataset size and taxonomic dataset

IPCO is based on covariance between the datasets. The size of the reference dataset and the taxonomy used will both affect the covariance between the taxonomic and functional datasets. IPCO was implemented at various taxonomic levels and various reference dataset sizes by subsampling the reference (10%, 30, 50, 70 and 80%).

Taxonomy was assigned to the representative sequences using the RDP database (v.11.4) [27] implemented in mothur (v.1.34.4) [28]. In addition, SPINGO [29] was used for species assignment using the RDP database (v.11.2). All levels of taxonomic classification were classified to a threshold of ≥80% confidence. At any level, if the classified threshold was below 80%, it was set as unclassified.

To ensure that the predictions are not due to the presence of homogenous samples and/or overfitting, the predicted profiles at each bootstrap were compared against shuffled wMGS sample profiles also (Labels on the R and L reference datasets were shuffled). This was carried out using the HMP stool KEGG and MetaCyc pathways and its paired OTU level taxonomic dataset at all reference/test split described above.

### Functional prediction with published methodology

For PICRUSt, the representative 16S rRNA sequences were mapped to Greengenes v13.5 to obtain closed representative OTU tables and processed through the PICRUSt pipeline to obtain KO profiles.

For Tax4Fun, the taxonomic information were assigned to the representative sequences provided in SILVA123 downloaded from Tax4Fun website. The OTU table with taxonomic profiles were processed through Tax4Fun R library to obtain KO profiles. All processing was carried out with default settings.

For Piphillin, OTU dataset and the representative OTU sequences were formatted as per requirement for Piphillin and all files were uploaded to the Piphillin website. An identity threshold of 90% was used and the KEGG database October 2018 version was used. The threshold of 90% was used as the default of 97% returned insufficient hits to reference genomes.

The KEGG pathway abundances were obtained using HUMAnN2 by processing the predicted KO profiles using the filtered HUMAnN1 KEGG legacy database. This allowed consistency in pathway calculation across different methods.

Sample-to-sample and feature-to-feature (KOs and KEGG pathways) abundance correlations were calculated by comparing the predicted values obtained from the various tools against the mWGS generated profiles using Spearman correlation. KOs and KEGG pathways present in both predicted and its paired mWGS datasets were considered.

### Comparison between IPCO at KO and KEGG level with published tools

The Sample-to-sample and feature-to-feature correlations observed using the published tools for all the sites were compared against the correlation observed for IPCO inferred KO and KEGG pathways using reference:test split of 50:50 at all sites. Further, the IPCO inferred KO profiles (faecal site, reference:test 50:50) at each bootstrap were processed using HUMAnN2 to obtain inferred-KO KEGG pathways which were correlated against its paired mWGS KEGG pathway profiles. This was done to investigate the accuracy of using the IPCO inferred KO profiles to determine functional abundance at KEGG pathway level.

### Comparison of inference capability from mWGS species and 16S species level as reference taxonomy on an external query dataset

It was investigated whether using mWGS species level dataset is sufficient as a reference set (Table L) to generate inferred functionality for the independent dataset. The independent dataset used is the ELDERMET community dataset [15]. If this generates comparable results as 16S taxonomic reference, then it would allow generation of a larger reference R and L table by adding more samples from curatedMetagenomicData hub which would incorporate more functionality else would have to use HMP or similar 16S dataset as Table L limiting the reference dataset size. Species level dataset were obtained for the 16S ELDERMET and HMP 16S as described earlier. IPCO was implemented with the R table as HMP pathways dataset, L table as HMP closed OTU or 16S species or HMP mWGS species dataset and Q table as ELDERMET 16S closed OTU or species dataset. Samples and common features from inferred table were correlated with the paired elder mWGS functional dataset.

The species and functional dataset for the 1180 healthy samples were obtained from curatedMetagenomicData. The selection of healthy samples is described in Ghosh et al. (submitted). This dataset is referred as reference healthy in the results. IPCO was implemented with R table as reference pathways, L table as species mWGS dataset and Q table as ELDERMET species 16S dataset. The inferred functionality dataset for the ELDERMET dataset was correlated with mWGS functionality dataset to obtain sample and feature correlations. Correlations obtained from using this healthy reference were compared with the correlation values from using HMP as reference.

### Validation with independent datasets

Validations of the IPCO’s prediction was carried out with the following assumptions: The healthy functional and taxonomic references from a particular site can be used to predict diseased samples also obtained from the same site without requiring diseased sample profiles as reference. This would confirm the appropriateness of using provided references. The second assumption is that the biological agreement between measured metabolite levels and mWGS functional profiles is reflected in the inferred functional profiles. To validate the results on external independent datasets, analyses were carried out on two cohorts: a colorectal cancer (CRC) publicly available data [30] and an in-house elderly community data [15]. The CRC dataset was used to validate the first assumption. The CRC cohort contains paired 16S and mWGS sequences obtained from faecal samples of healthy and CRC individuals. The mWGS taxonomic and functional profiles were obtained from curatedMetagenomicData for the healthy (*n* = 50) and CRC samples (*n* = 41) based on the disease stratification provided. The forward reads from the 16S dataset were quality filtered using Trimmomatic. The quality filtered reads were processed using USEARCH and taxonomically annotated as described earlier. Predicted functional profiles (KEGG pathways) of the 16S CRC samples were also obtained for PICRUSt, Tax4Fun and Piphillin as described earlier.

At first, it was assessed whether IPCO can be applied to infer the functional potential from the 16S microbiota profiles of diseased samples or not using the CRC cohort. The 16S genus level and mWGS pathways (KEGG and MetaCyc) profiles of the CRC samples were used as reference in IPCO for inferring functionality of the queried 16S CRC samples. This was carried out using the bootstrap approach (100 iterations) described earlier to ensure the reference and test datasets (reference/test split 50:50) do not contain the same CRC samples. This would serve as the baseline for all other comparisons where the applicability of the healthy samples as reference would be determined. Next, using the taxonomic and paired functional datasets of the healthy samples from the same cohort as reference for IPCO, functionalities were inferred for the 16S genus profiles of the CRC samples. Genus level reference dataset was used as a mediator as most 16S datasets are usually classified down to genus level. Both 16S and mWGS genus dataset from the in-cohort healthy were used as reference. Finally, the external healthy taxonomic and functional profiles provided in IPCO were used as reference to infer the functionality of the 16S CRC samples. The healthy samples from the CRC samples were also present in the IPCO healthy reference and were removed before implementing IPCO. Using multiple reference datasets with IPCO methodology allowed comparing the differences in predictive capacity observed with using different reference datasets (that includes in-cohort and external data). Further, functionalities predicted for 16S CRC samples using PICRUSt, Tax4Fun and Piphillin were also included to compare the differences in prediction observed in different tools. Correlation observed (Sample-to-sample and feature-to-feature) using the CRC samples as reference vs healthy samples as reference were compared for IPCO to determine the appropriateness of IPCO’s reference. Correlation observed (Sample-to-sample and feature-to-feature) between predicted functional profiles obtained using 16S CRC samples and mWGS CRC samples using published tools were also compared with IPCO to evaluate the performance of all the tools.

The elderly cohort contains mWGS, 16S, and metabolome datasets for the same samples. This was used to validate the second assumption: biological agreement between metabolite profile and mWGS functional potential (KEGG and MetaCyc pathways) is replicated in inferred functional profiles also. To investigate the biological signal in the predicted functionalities, functional profiles were obtained from all tools.

For IPCO, paired functionality (KEGG pathway and MetaCyc) and taxonomy at species level from the reference healthy datasets provided in IPCO was used to inferred functionalities for the 16S elderly samples. Functional profiles (KEGG pathways) were obtained using PICRUSt, Tax4Fun and Piphillin using the same approach as described earlier for the elderly community 16S dataset. Two types of metabolites: bile acids and short chain fatty acids (SCFAs) which are widely studied in microbiome research were considered for investigation. The metabolite dataset was log10 transformed on the measured metabolite level after adding 1e-05 as minimum count value. The mWGS functional profiles were correlated with the metabolite profiles and the directionality, degree of association and significance was noted. Significance of mWGS profile correlation was determined by p-adjusted ≤0.05 unless stated otherwise. The inferred functions obtained from all the tools including IPCO were then correlated with the same metabolites and the results were compared with mWGS results to investigate the direction, correlation, and significance (*p*-value ≤0.1). Only key pathways responsible for these metabolites were considered and agreement with mWGS results in terms of directionality and significance were considered to be correct, with a change in directionality or non-significance being considered as false positives.

### Statistical analysis

All analysis was carried out in R (v.3.5.1) [31]. All correlations measured were carried out using Spearman correlation. Kruskal-Wallis test was used as applicable. Dunn’s test using dunn.test library (v.1.3.5) [32] was used for pairwise comparison at different taxa and sample threshold levels. *P*-value adjustment was carried out using Benjamini-Hochberg procedure. Covariance between paired taxonomic and functional dataset was investigated with co-inertia analysis using ade4 (v.1.7.13) library [33]. Significance of co-inertia was determined with the ade4 randtest function. Plots were created using ggplot2 (v.3.1.0) [34], RColorBrewer (v.1.1.2) [35] and gridExtra (v.2.2.1) [36] R libraries.

## Supplementary information

**Additional file 1: **Supplementary figures**.** This file contains all the supplementary figures, their title and legends referenced in the main manuscript.

**Additional file 2: **Supplementary tables**.** This file contains all the supplementary tables containing the *p*-values, RV-coefficient, correlation values, their title and legends referenced in the main manuscript.

## Data Availability

R library, data and documentation are available at https://github.com/IPCO-Rlibrary/IPCO. All reference datasets are obtained from curatedMetagenomicData R library and HMP1 project. **Project name:** IPCO-Rlibrary. **Operating system(s):** Platform independent. **Programming language:** R. **Other requirements:** ade4 R library. **License: ≥** GNU version 2. **Restrictions for use by non-academics.** None.
