# Supplementary material for: IPCO: Inference of Pathways from Co-variance analysis
Source: BMC Bioinformatics. 2020 Feb 18;21:62. doi: 10.1186/s12859-020-3404-2 (PMC7029613; doi:10.1186/s12859-020-3404-2)
Supplement: Supplementary file 1 — Additional file 1: Supplementary figures. This file contains all the supplementary figures, their title and legends referenced in the main manuscript. [file 12859_2020_3404_MOESM1_ESM.docx]

**Supplementary figure 1** Sample-to-sample and feature-to-feature correlations between inferred and mWGS functional profiles obtained for MetaCyc pathway schemes using different transformation methodologies


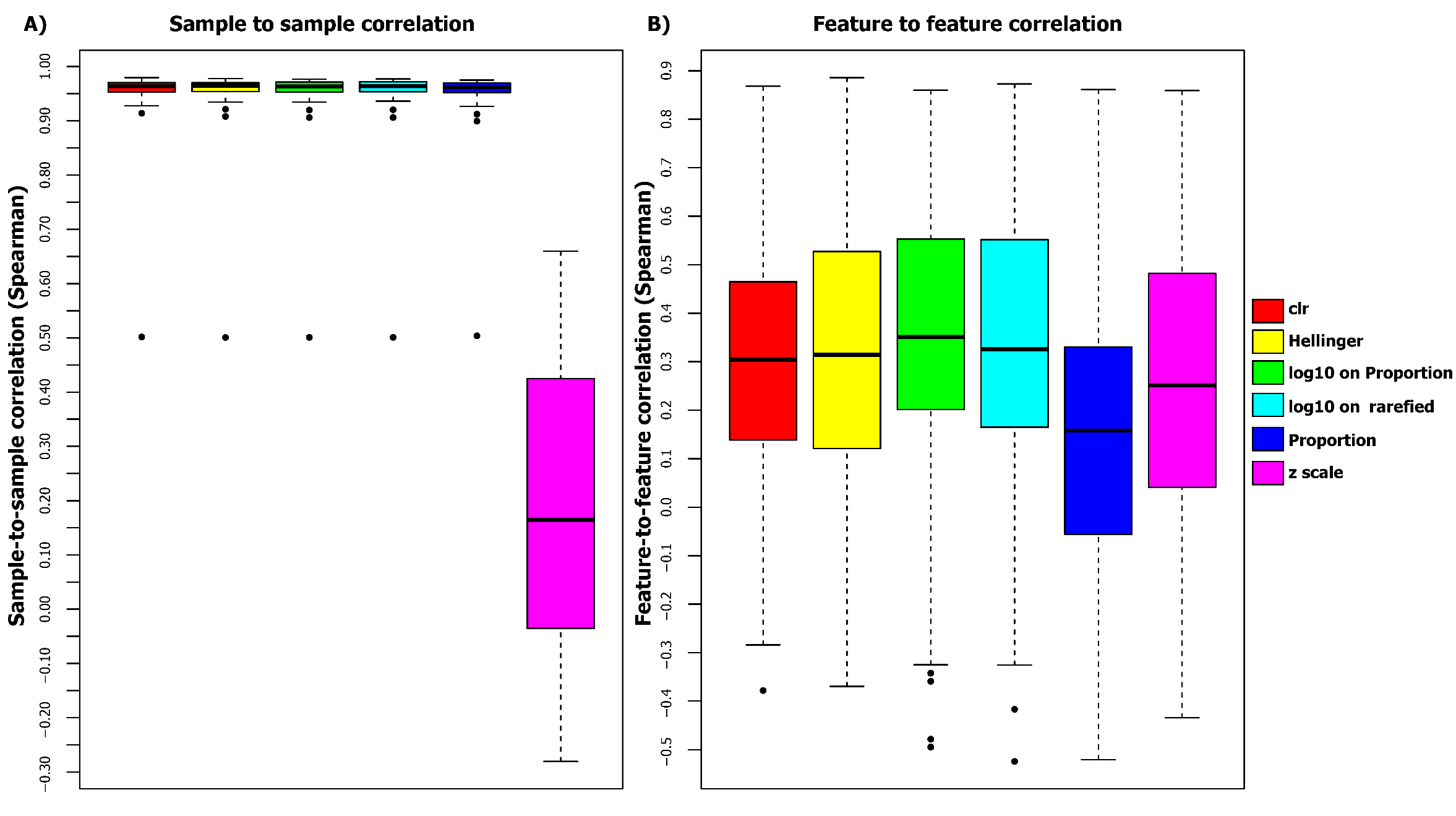
 The effect of transformation/normalisation methodologies on preliminary analysis of IPCO predictions using the MetaCyc functional profile datasets, in terms of **A)** sample to sample correlations and **B)** the feature to feature correlations for IPCO inferred sample and functional profiles to mWGS sample and functional profiles

**Supplementary figure 2** Comparison of sample-to-sample and feature-to-feature correlations obtained between the inferred and the mWGS MetaCyc pathway abundances at different taxonomic levels and reference dataset size


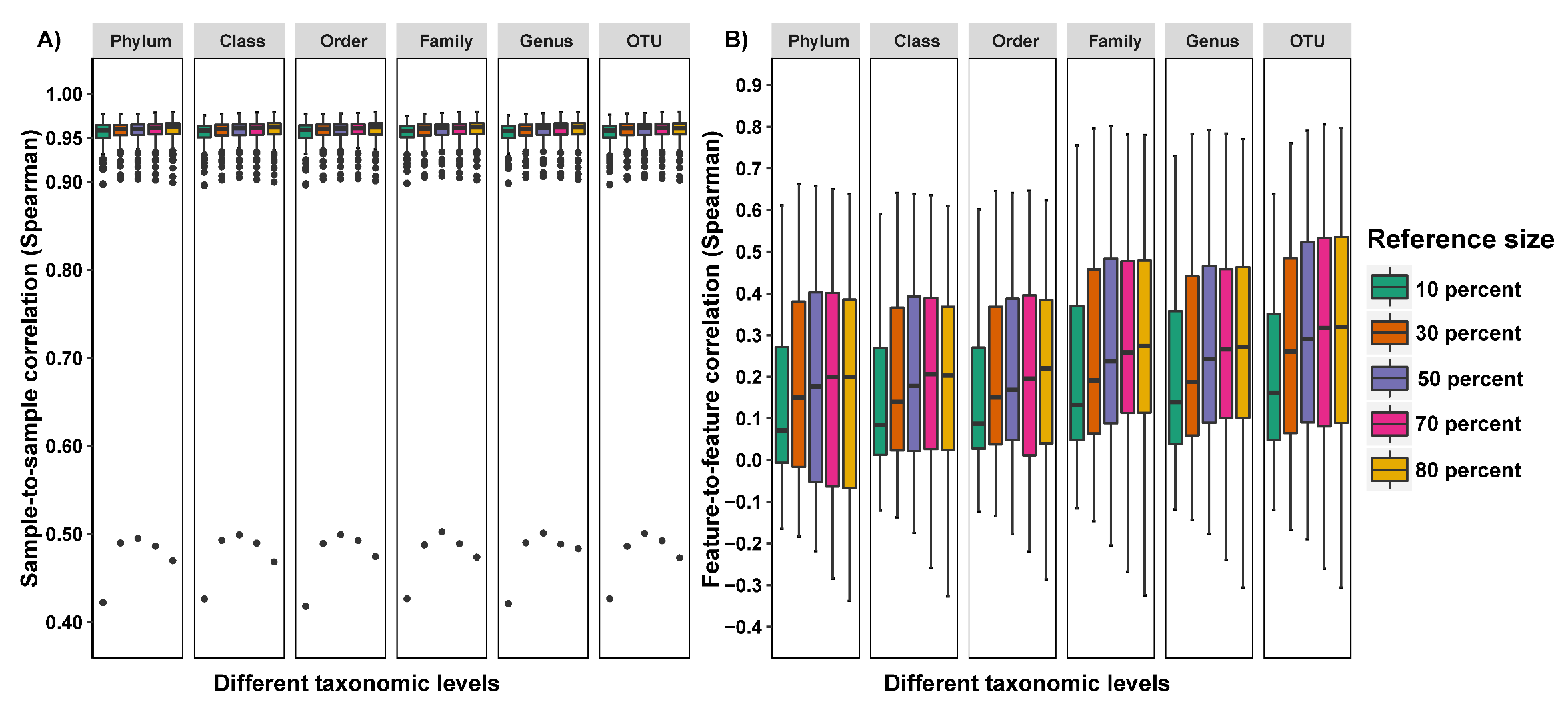


IPCO’s prediction of MetaCyc pathways abundance varies with reference dataset size and taxonomic level **A)** Sample to sample correlations across reference size and taxonomic levels. **B)** The feature to feature correlation values of features improve with larger reference datasets and with lower taxonomic level

**Supplementary figure 3** Comparison of mWGS profiles against inferred profiles (KEGG and MetaCyc) from query data (Q) obtained from shuffled reference datasets (R and L)


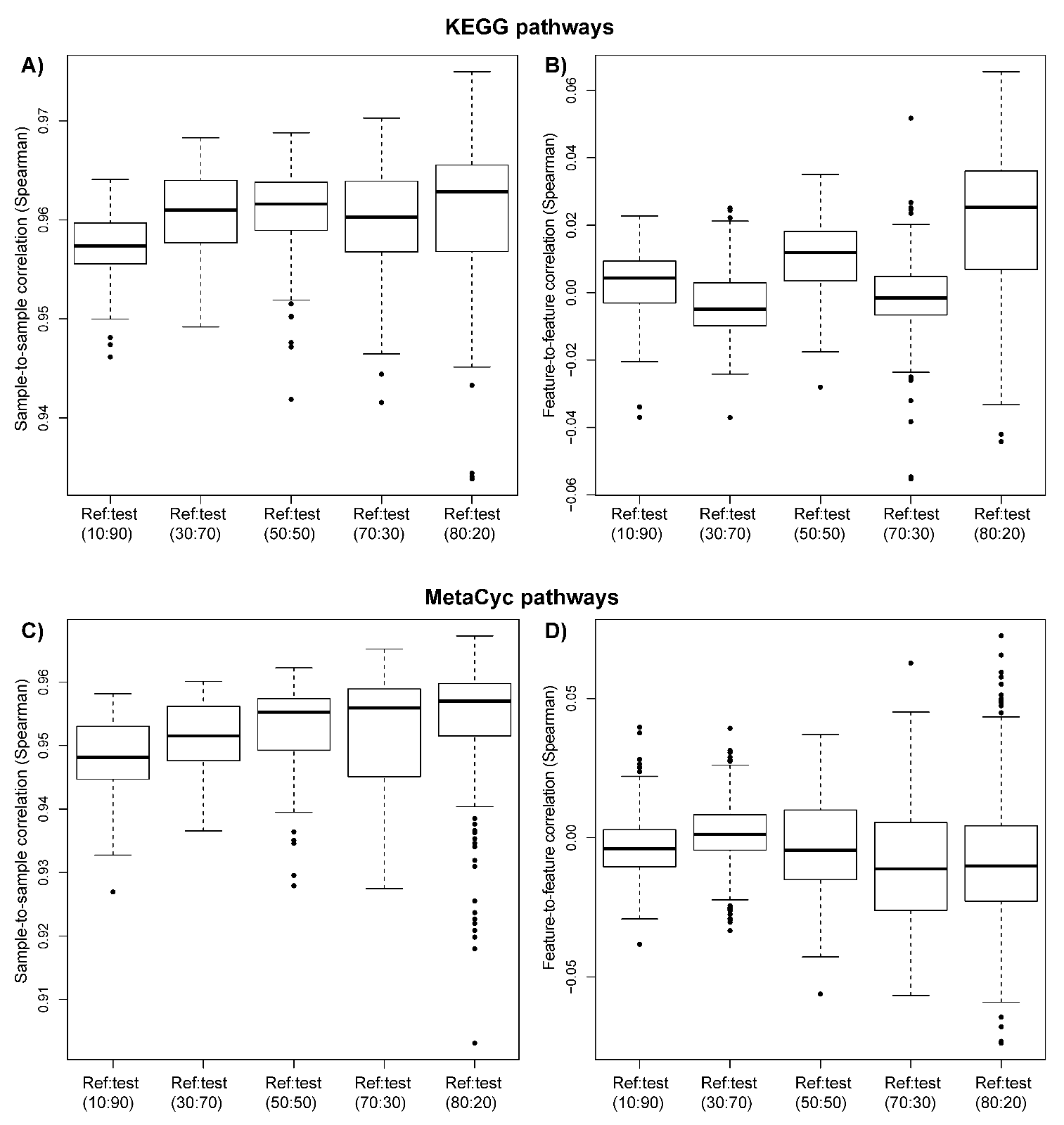


Boxplots showing correlation values for inferred samples and features against mWGS pathway profiles. Figure 3A-B shows the sample and feature correlations observed with the KEGG pathways profiles when the query sample labels are shuffled. In figure 3C-D, the sample and feature correlation observed between predicted and mWGS MetaCyc profiles

**Supplementary figure 4** Evaluating the correlation between IPCO inferred-KO KEGG pathways against the mWGS KEGG pathways


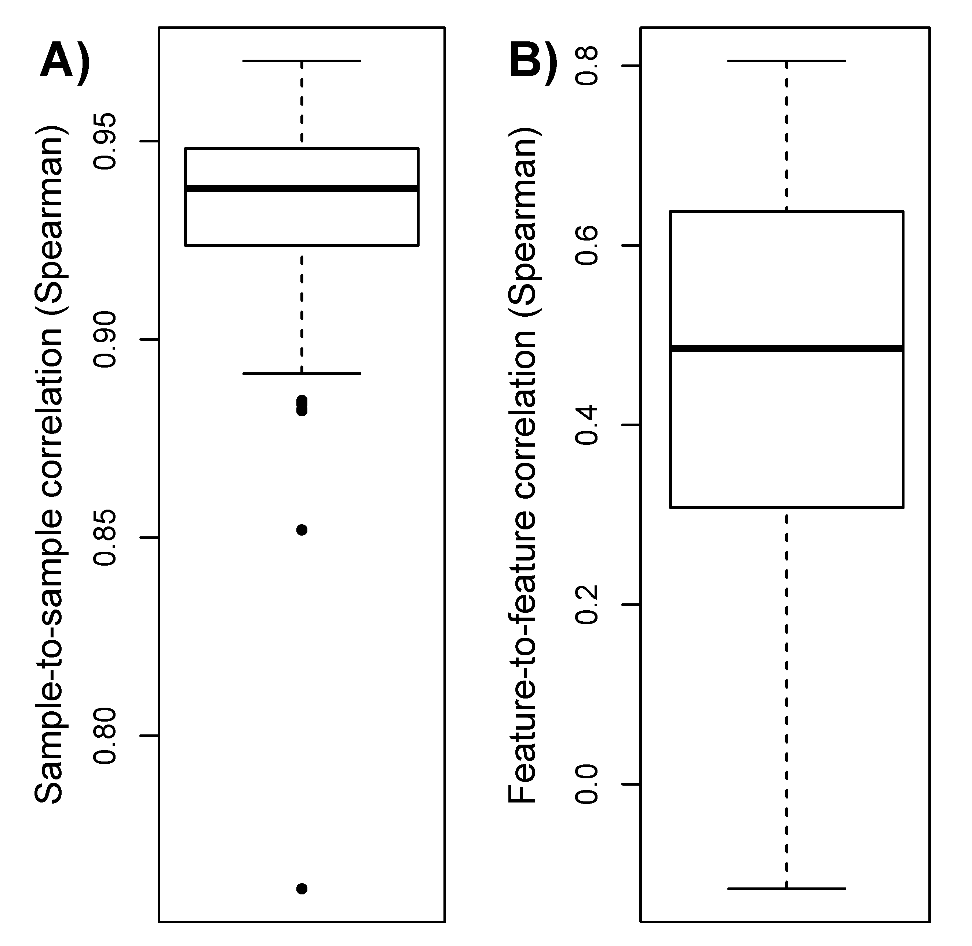


Spearman correlation of KEGG pathways calculated using HUMAnN2 from the IPCO inferred KO profiles compared to mWGS KEGG pathways. A) Sample-to-sample correlations observed and B) Feature-to-feature correlations observed.

**Supplementary figure 5** Feature correlation vs Mean coverage obtained from published tools


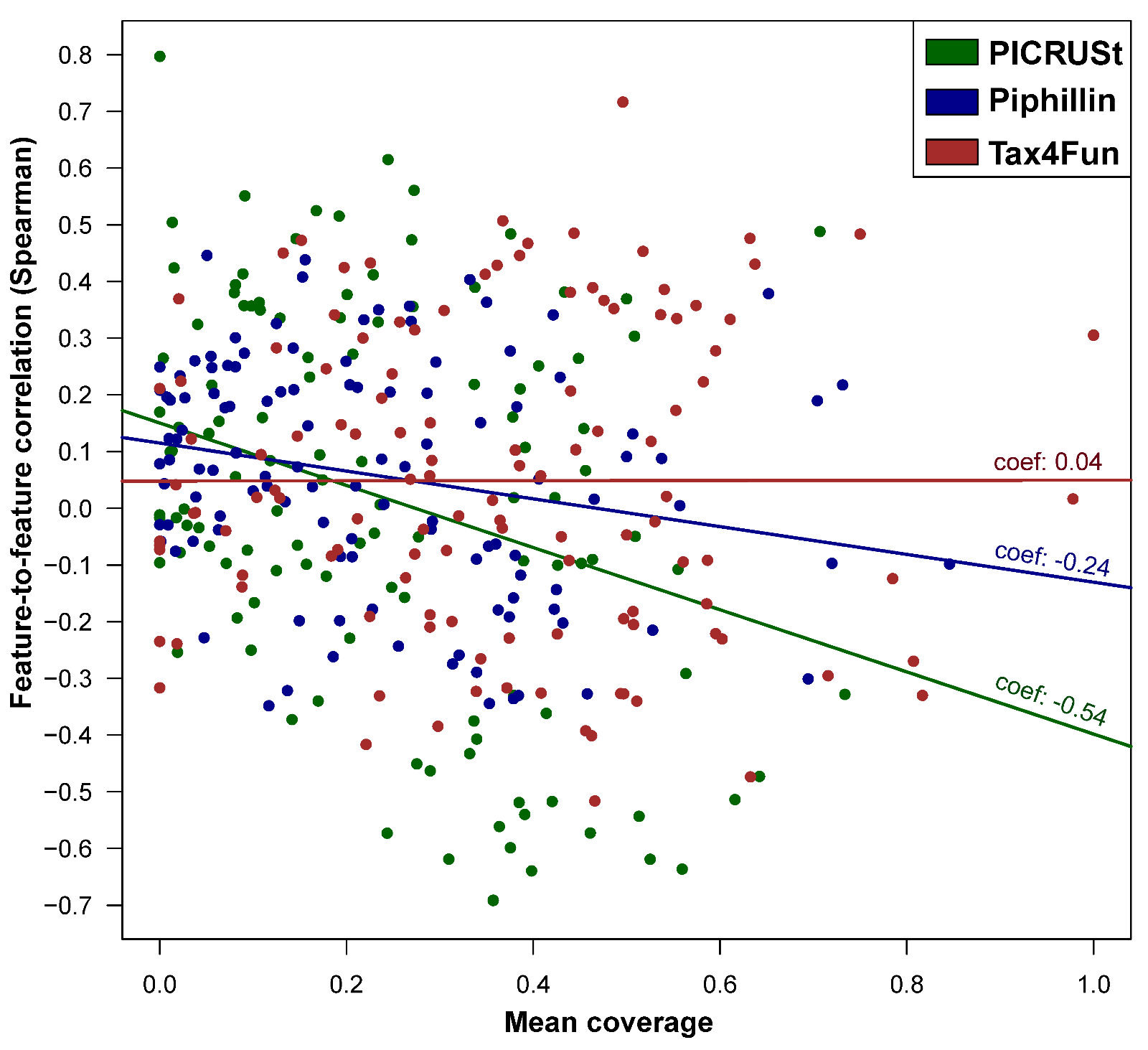


A scatter plot of correlation values obtained from feature-to-feature correlation (KEGG pathways) vs mean coverage obtained from the published tools. The coefficient of correlations are reported for the three tools along with the predicted line.

**Supplementary figure 6** Comparison of using 16S and mWGS taxonomic dataset as table L when inferring for external dataset


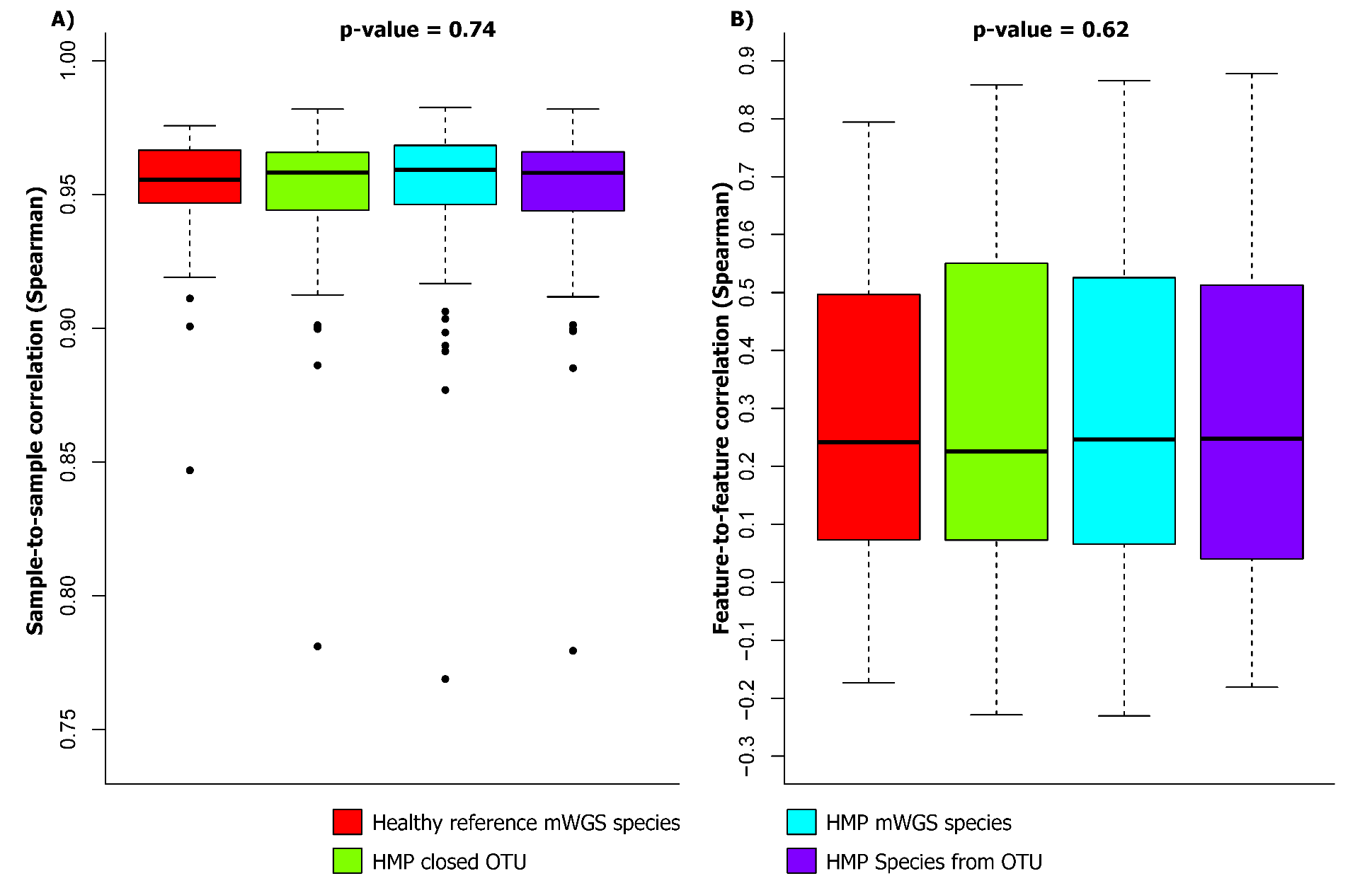


Supplementary figure 6 shows sample and feature correlation of inferred MetaCyc pathways of elderly 16S dataset obtained from using HMP 16S species and closed OTU, HMP mWGS species and healthy reference mWGS species as reference taxonomic dataset (table L). No significant change in observed sample (**A**) and feature (**B**) correlation for the external dataset when using the closed OTU level or species level dataset derived from either 16S representative sequences or mWGS taxonomy (**table L**). The external dataset (**table Q**) is collapsed to closed OTU level or species level derived from 16S representative sequences

**Supplementary figure 7** Accuracy of using different references to predict MetaCyc profiles for CRC samples


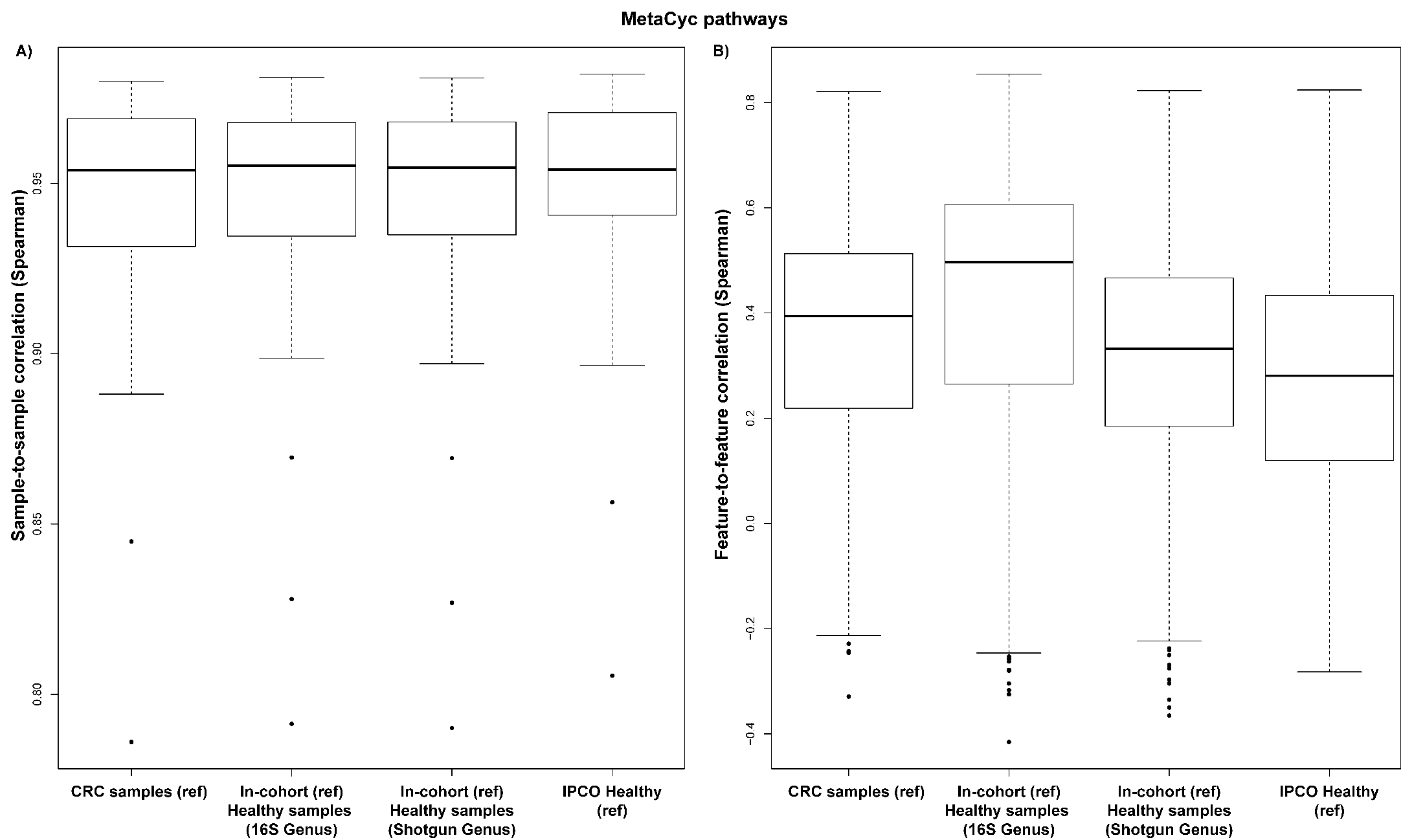


Supplementary figure 7 highlights the sample (A) and feature (B) correlation obtained when using different samples types as reference taxonomy and functional datasets to predict MetaCyc profiles from 16S genus level of only CRC samples.

CRC samples (ref): Using the CRC samples as references, In-cohort (ref) Healthy samples (16S Genus): Using the healthy samples from the same cohort with 16S Genus profiles as reference taxonomy, In-cohort (ref) Healthy samples (Shotgun Genus): Reference is healthy samples from the same cohort with refernce mWGS Genus profiles, IPCO Healthy (ref): The healthy samples provided with IPCO as references after excluding Zeller *et al.* samples
